# Current Development in Biomaterials—Hydroxyapatite and Bioglass for Applications in Biomedical Field: A Review

**DOI:** 10.3390/jfb13040248

**Published:** 2022-11-16

**Authors:** Diana Georgiana Filip, Vasile-Adrian Surdu, Andrei Viorel Paduraru, Ecaterina Andronescu

**Affiliations:** 1 Department of Science and Engineering of Oxide Materials and Nanomaterials, Faculty of Chemical Engineering and Biotechnologies, University Politehnica of Bucharest, 060042 Bucharest, Romania; 2 National Centre for Micro and Nanomaterials, University Politehnica of Bucharest, 060042 Bucharest, Romania; 3 Academy of Romanian Scientists, 50085 Bucharest, Romania

**Keywords:** bioceramics, biomaterials, 45S5 Bioglass^®^, hydroxyapatite

## Abstract

Inorganic biomaterials, including different types of metals and ceramics are widely used in various fields due to their biocompatibility, bioactivity, and bioresorbable capacity. In recent years, biomaterials have been used in biomedical and biological applications. Calcium phosphate (*CaPs*) compounds are gaining importance in the field of biomaterials used as a standalone material or in more complex structures, especially for bone substitutes and drug delivery systems. The use of multiple dopants into the structure of *CaPs* compounds can significantly improve their in vivo and in vitro activity. Among the general information included in the Introduction section, in the first section of this review paper, the authors provided a background on the development of hydroxyapatite, methods of synthesis, and its applications. The advantages of using different ions and co-ions for substitution into the hydroxyapatite lattice and their influence on physicochemical, antibacterial, and biological properties of hydroxyapatite are also presented in this section of the review paper. Larry Hench’s 45S5 Bioglass^®^, commercially named 45S5, was the first bioactive glass that revealed a chemical bond with bone, highlighting the potential of this biomaterial to be widely used in biomedicine for bone regeneration. The second section of this article is focused on the development and current products based on 45S5 Bioglass^®^, covering the historical evolution, importance of the sintering method, hybrid bioglass composites, and applications. To overcome the limitations of the original biomaterials, studies were performed to combine hydroxyapatite and 45S5 Bioglass^®^ into new composites used for their high bioactivity and improved properties. This particular type of combined hydroxyapatite/bioglass biomaterial is discussed in the last section of this review paper.

## 1. Introduction

Even if numerous researchers are actively working to improve bioceramics functions, they have not yet established a definition of bioceramics. According to the Consensus Conference of the European Society of Biomaterial—Chester, England (1986), biomaterials are “any substances, other than a drug, or combination of substances, synthetic or natural in origin, which can be used for any period of time, as a whole or as a part of a system which treats, augments, or replaces any tissue, organ or function of the body”. Taking into consideration this definition, different types of ceramics used in medical and dental fields for the human body are classified as bioceramics. Some opinions are still different for bio-technology-related ceramics, such as immobilized ceramics, carriers of enzyme, and ceramics devices that are used for the separation or purification of proteins [1].

**Figure 1:**
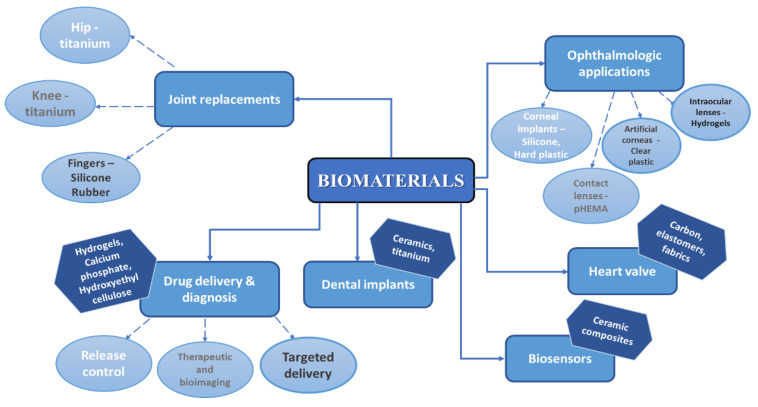
Schematic diagram of different categories of biomaterials and their applications [8].

Biomaterials are a perfect alternative for conventional materials used in numerous biomedical applications as they manifest a low or negligible toxicity in relation to humans, other organisms, and to the environment. Applications of different groups of bioceramics include the replacement, repair, or regeneration of bone or teeth and coating of metallic implants [2,3,4,5] but is not limited to this. Hench and Wilson states that biomaterials are bioinert, tolerable, bioactive, and resorbable [6] and due to their interactions with bones or soft tissue, once inside the human body, biomaterials may form direct chemical bonds and accelerate the healing process and/or improve device adherence [7]. Without doubt, the most explored areas of biomaterials research are tissue engineering, drug delivery, and bone regeneration. In Figure 1, a schematic diagram containing various applications of biomaterials is presented [8]. 

It is well known that almost 70% of human bone consists in the calcium phosphate (*CaP*) mineral, and as a consequence, *CaPs* biomaterials are the first choice for repairing damaged bones. The advantages of *CaP* biomaterials are the following: low cost, stability, safety, and simple certification for clinical use. Even if the first commercial *CaP* bone graft substitutes were made available 40 years ago, in the past years a massive development was made to extend the use of *CaP* in the biomedical field, as carrier for ion delivery, for biologically active agents, and in the field of biomineralization [9]. Recent studies are focused on nearly inert bioceramics and bioactive bioceramics (and biodegradable) [10,11] and bioglass and hydroxyapatite are part of this category [12].

Hydroxyapatite (*HAp*, molecular formula Ca_10_(PO_4_)_6_(OH)_2_) is the main inorganic constituent of human hard tissues [13] and due to its osteogenic, osteoconductive, and osteoinductive properties, is frequently used as synthetic cement for bone and dental reconstruction [14,15]. The porous surface and biodegradable properties enables *HAp* to be successfully used in chemotherapy and antibiotic drug delivery systems [16,17,18,19].

In 1971, Larry Hench published a peer research paper in which he presented the development of a bone-bonding calcia-phosposilicate glass–ceramic [20]. This material is now commercially named Bioglass^®^ [21], a particular type of bioactive glass with Na_2_–CaO–SiO_2_–P_2_O_5_ structure [22]. The evolution of bioactive materials used for implants and repair or replacement of bones is based on the discovery made by Hench. The current global situation has increased public consciousness on the importance of having the best protocols to prevent the spread of microorganisms. The development of novel antimicrobial biomaterial coatings and surfaces has gained attention [23]. 

In this review paper, the authors summarized recent and relevant studies on hydroxyapatite and Bioglass. According to numerous studies, the substitution of various types of ions into hydroxyapatite and bioglass lattice can increase the performance of the biomaterial and can positively influence the applications, aspects that are furthered investigated in this paper. 

## 2. Focus on Hydroxyapatite 

Biomaterials are traditionally used in the medicine field for different types of implants [24,25,26], but with recent scientific and technological developments, the field of biomaterials has grown, and new improved materials are widely studied for different applications. Calcium phosphates (*CaPs*) are more biocompatible than many other categories of ceramics and inorganic nanoparticles. Due to their biocompatibility and variable stoichiometry, surface charge density, dissolution properties, and functionality, *CaPs* are suitable to be used as drug and growth factor delivery [18]. Hydroxyapatite is also used in multiple non-medical sectors, such as chemical sensors for liquid chromatography column [27], gas sensors, catalysts, [28] or in wastewater treatment [29]. Calcium phosphates exists in different forms, depending on temperature, impurities, and the presence of water molecules [30]. The bioactivity properties and degradation behavior are determined by Ca/P ratio, crystallinity, and phase purity [30,31,32]. 

### 2.1. Structure of Hydroxyapatite

**Figure 2:**
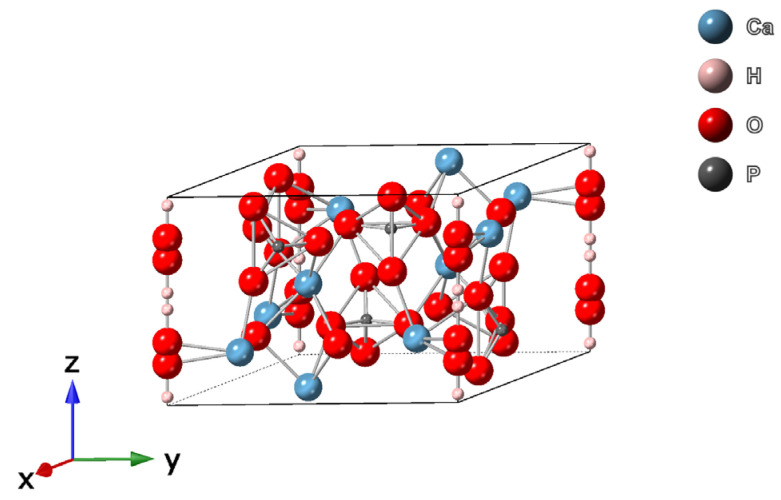
The molecular structure of hydroxyapatite—unit cell perspective of hexagonal crystal structure with P6_3_/m symmetry [36,37].

Hydroxyapatite has a complex chemical structure, with Ca/P ratio of 1.67, a hexagonal symmetry S.G. P6_3_/m with lattice parameters *a* = 0.95 nm and *c* = 0.68 nm [19,33], and it is the most stable calcium phosphate salt at normal temperatures and pH between 4 and 12 [34]. Ca^2+^, PO_4_^3−^, and OH^−^ ions are distributed over 2 symmetric parts of the unit cell and each unit cell of *HAp* contains 10 Ca^2+^ atoms distributed in 2 sites, 6 PO_4_^3−^ ions in the tetrahedron, and 2 OH^−^ ions surrounding the Ca^2+^ ions at the corners of the unit cell (Figure 2). The apatite structure has a carbonate group in two positions: the OH^−^ sub-lattice generating type A carbonate apatite or the [PO_4_]^3−^ sub-lattice generating type B-apatites. When high temperature is used in the synthesis process, type A apatites are obtained [35].

### 2.2. Synthesis of Hydroxyapatite

Expected performance of the material, economic aspects, precursors, type of solvent used, and the necessary surfactant material, but also the distribution of particles, the need of complex expensive additional steps, and the possibility of agglomerations or impurities in the crystalline structure, are taken into consideration when choosing a synthesis method. Most researchers focused their studies on controlling these parameters by developing new routes of synthesis or by modifying existing routes [38]. 

*HAp* can be obtained using a variety of methods, such as sol–gel, chemical precipitation, polyol, hydrothermal, sonochemical and microemulsion, solid-state method, or combustion and thermal decomposition. Recently, due to the attention on environmental issues, innovative methods of synthesis were developed, such as biomimetic methods or the use of hydrothermal molten salt [39]. Depending on the method of synthesis, *HAp* particles can have different sizes and shapes, as presented in Table 1.

The possibilities of synthesis *HAp* can be classified as:

- Wet–chemical synthesis: precipitation, hydrothermal, and sol–gel method;
- Dry–chemical synthesis: solid-state reactions and mechanochemical method;
- High-temperature methods: combustion, spray pyrolysis, and thermal decomposition;
- Use of bioresources: animal sources (sheep, pig, and goat teeth and bones); marine sources (bones, red algae, fish scale, corals, or seashells); and plant sources (bamboo, potato orange banana peels, calendula flowers, etc.).

### 2.3. Substitution of Various Ions in HAp

**Figure 3:**
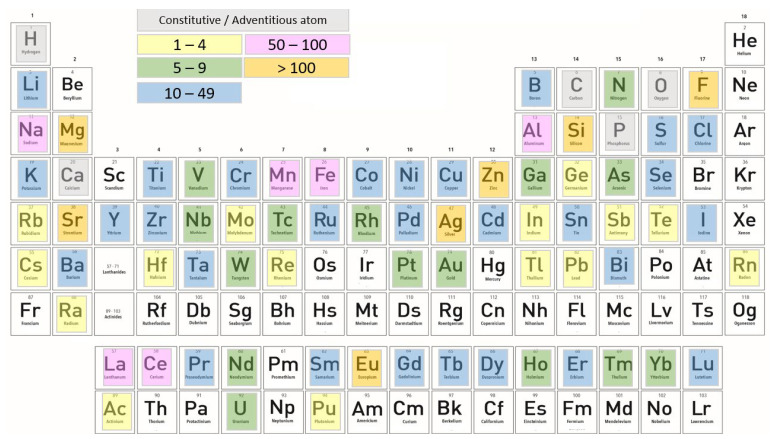
Periodic table of elements—each element has a corresponding color (yellow, green, blue, pink, and orange) which suggests the total number of its literature reports. The only adventitious element is carbon because most HAp crystals precipitated under the atmospheric conditions contain finite amounts of the carbonate ion. For this statistic, Scopus and Google Scholar search engines were used [66].

*HAp* has an excellent biocompatibility, but the bioactivity can be enhanced using substitution ions in its unit cell [62]. One disadvantage of synthetic stoichiometric *HAp* is having poor mechanical properties, and thus is not indicated to be used as a standalone material [63]. *HAp* is able to accept various compositional structures in its sub-lattices, and depending on which substitution ions are used, a new *HAp* composite with improved properties for specific biomedical applications can be obtained [64,65]. If we consider the evolution of trends in the type, frequency, and competition of dopants used, it can be observed that 72 elements of the periodic table were successfully incorporated into its structure. The overall use of individual chemical elements as dopants in *HAp* is presented in Figure 3 [66]. Chemical elements are marked with different colors, depending on the number of studies that were published with doped hydroxyapatite. The number of studies can be found in the legend. 

With a flexible hexagonal structure, *HAp* can be substituted with numerous cationic ions (Sr^2+^, Na^+^, K^+^, Mg^2+^, Zn^2+^, Ag^+^, etc.) and anionic ions (CO_3_^2−^, SiO_4_^4−^, SeO_3_^2−^, F^−^, Cl^−^, Br^−^, and others) [67]. The shorter distance from Ca II sites and the Ca II-O bond makes cations with smaller size than Ca atoms to be preferred for substitution [68,69]. It has been proven that after substitution, *HAp* will have the same hexagonal structure, but the minor internal tensions can cause changes in thermal stability, solubility and hardness. The use of various doping ions that enter into the structure of hydroxyapatite can enhance mechanical performance and also improve multiple biofunctionalities, such as osteogenesis, resistance to adhesion, and proliferation of bacterial strains [70].

#### 2.3.1. Cationic Substitutions

Cationic substitutions are performed by replacing Ca^2+^ ions from hydroxyapatite’s lattice with monovalent ions (lithium—Li [71], sodium—Na [72], silver—Ag [73,74]), bivalent ions (magnesium—Mg [75], strontium—Sr [76], zinc—Zn [77]) or multivalent ions (aluminum—Al [78], cerium—Ce [79], gallium—Ga, or titanium—Ti [80]). The cationic substitution has been extensively studied, but it has been shown that not all the elements can be used for improved biofunctional properties. The advantages of using Zn, Mg, and Sr is that these ions have the capability to increase the osteogenic capacity. Co has angiogenic activity, and the use of Ce, Zn, and Ag cations for substitution offers excellent antimicrobial properties.

Multiple ions can be used for cationic substitution. From all the elements, Magnesium is one of the key elements because human bones contain about 60% of Mg^2+^ so it has an essential role in the health of teeth and bones. Magnesium ions have the potential to influence bone metabolism, cell adhesion and proliferation, and a deficiency in Mg^2+^ can cause bone fragility, stoppage of bone growth, and osteopenia [81,82]. Mg-doped hydroxyapatite can be synthesized by an aqueous precipitation method [83,84], but the use of hydrothermal technology was also reported [85]. As published by Matsunaga [69], the formation energies of smaller ions in hydroxyapatite structure decreases with the increase in pH of the solution. Ca^2+^ are released from the hydroxyapatite lattice at lower values of pH, and the effect is in reverse with the increase in pH values. If smaller size cations are replacing the Ca^2+^ atoms, the adjacent O^2−^ atoms tend to move to substitute cations because of the difference in ion size. Once the Ca^2+^ ions are substituted by Mg^2+^ ions, the unit cell volume will decrease and the result is a smaller ionic radius of 0.072 nm. Various secondary products are formed with the synthesis of Mg-doped hydroxyapatite, such as Mg(OH)_2_, brushite at higher degrees, or β-tricalcium phosphate [86]. The formation of *CaP* as secondary products is the result of the limited degree of substitution of magnesium ions in hydroxyapatite structure. Besides the beneficial effects of substitution of Mg in the *HAp* lattice related to bone structure, antibacterial properties against *E. coli, P. aeruginosa, S. aureus,* and *C. albicans* were also demonstrated [75,87].

Due to their smaller radius of 0.074 nm, Zn^2+^ ions are theoretically more appropriate to be used for the substitution of hydroxyapatite. Zn-doped hydroxyapatite is suitable to be used in biomedical field due to the effects of Zn^2+^ on metabolic processes. Generally, a precipitation method at room temperature [88] or at 60–90 °C [89] is used, but there are also studies which reported the use of other technologies, such as ion exchange, hydrothermal synthesis, and the sol–gel method [90]. The crystallinity of hydroxyapatite is reduced when substitutional ions with smaller size are used. The explanation may be that the ions are partially incorporated, leading to the inhibition of crystal growth. The antibacterial properties of Zn-doped hydroxyapatite increase with the increase in Zn^2+^ ions content. *Zn/HAp* biomaterial can be used on stem cell differentiation and because of the antibacterial properties, the biomaterial might be used as a coating on metal implants [91].

Some studies have demonstrated that Fe^2+^/Fe^3+^ ions can improve tissue regeneration, but the deficiency of Fe ions could contribute to the inhibition of osteoblast cell mineralization [92]. Fe^3+^ substitution in *HAp* structure leads to a decrease in particle size and an increase in hardness and dielectric properties for *HAp*. Similarly, this biomaterial is suitable for drug release systems, is blood-compatible in nature, and has an increased bioactivity. Fe-doped hydroxyapatite is obtained using precipitation, hydrothermal, and reflux methods, without the formation of other crystalline phases. Antibacterial tests suggest that Fe-substituted *HAp* is strongly active against bacterial strains and can be used for antibacterial applications [93].

It is well known that lanthanides have high bioactivity and the ability to substitute calcium ions in molecules due to their narrow emission bands and long emission lifetime. Lanthanides are widely used for magnetic resonance imaging and as luminescent probes for biosensors for in vivo imaging applications [94]. Even if they are less studied than other chemical elements, Cerium ions possesses excellent antioxidant properties against oxidative stress-induced cell damage and Ce-doped *HAp* nanomaterials have no cytotoxic effect. Therefore, the Ce-doped nanomaterials could be used for cellular imaging [95]. Due to its two forms, Ce^3+^ and Ce^4+^ have the unique property to switch between oxidation states and recent studies have shown that the reduction of CeO_2_ from Ce^4+^ to Ce^3+^ happens due to the formation of oxygen vacancies without changing its own structure [96]. Among the numerous techniques for preparation of Ce-doped hydroxyapatite, researchers mostly used the co-precipitation method [79], hydrothermal technique [97], sol–gel method [98], microwave irradiation technique [99], and ultrasonic-assisted precipitation synthesis [98]. Sol–gel preparation is the preferred method due to its simplicity, reduced additional costs, ease of handling, requirement of lower temperature, and the high purity and crystallinity of Ce-doped *HAp* particles [100,101].

#### 2.3.2. Anionic Substitutions

In contrast with monovalent anions (F^−^ or Cl^−^) that can substitute OH^−^ groups, bivalent anions (HPO_4_^2^, SO_4_^2−^, SeO_3_^2−^, CO_3_^2−^) are able to substitute PO_4_^3−^ groups with formation of hydroxide and calcium vacancies. As already evidenced in several studies, carbonate ions are naturally present in biological apatite structure in 4–8 wt.% [102]. The CO_3_^2−^ group can partially replace both PO_4_^3−^ groups in the B sites of the hydroxyapatite structure, leading to B-type substitution, or the hydroxyl ion, leading to A-type substitution. The B-type substitution is found in the bones of most species, where the A/B ratio is in the range 0.7–0.9. For A-type substitution, the experimental procedure should be performed at a temperature of 800 °C–1000 °C in a dry CO_2_ atmosphere. B-type substitution is mostly obtained by wet precipitation under atmospheric conditions. A combined A/B-type substitution was also identified when two CO_3_^2−^ ions replace one hydroxyl group and one phosphate group. As a source of carbonate ions, sodium hydrogen carbonate (NaHCO_3_) [103,104], di-ammonium carbonate ((NH_4_)_2_CO_3_) [105], or calcium carbonate (CaCO_3_) can be used [106]. The substitution can be carried out by multiple routes, such as wet chemical precipitation, emulsion methods, co-precipitation, mechanochemical methods, sol–gel synthesis, or microwave precipitation [107]. Carbonate-substituted hydroxyapatite can potentially be used in bone regeneration due to its effect on cell proliferation compared with stoichiometric *HAp* and the effect of the synthesized material depends on the percentage of CO_3_^2−^ substitution [108,109]. In some in vitro studies, it was reported that the production of collagen by human osteoblast cells was increased by carbonated hydroxyapatite, compared with pure hydroxyapatite. This is related to the production of type I collagen, higher in cell culture medium with carbonated hydroxyapatite, depending on extracellular calcium concentration [110].

#### 2.3.3. Effects of Co-Doping on Physicochemical, Antibacterial, and Biological Properties of HAp

Co-doped hydroxyapatite can be synthesized from synthetic precursors and/or from biogenic sources. Ag^+^, Zn^+^, Cu^2+^, and F^−^ act as antibacterial agents; M^g2+^, Sr^2+^, and Mn^2+^ improve bone metabolism; and SeO_3_^2−^, SeO_4_^2−^, and Fe^3+^ have anti-cancer properties. The type, size, and concentration of ionic substitution agents can lead to changes in the microstructure, grain size, thermal stability, lattice parameters, crystal morphology, crystallinity, and solubility of *HAp*.

The substitution of Zn^+^/Cl^−^ ions in the HAp lattice using the precipitation method increased the degree of crystallinity in comparison with pure *HAp*. It was demonstrated that Zn atoms occupy the interstitial sites in hydroxyapatite and does not substitute Ca atoms in Ca(I) or Ca(II) sites. Slight changes in unit-cell volume were noted when the Zn^2+^ cation is placed in site *2b* in *HAp*, due to the increase in the *c* lattice parameter and the decrease in the *a* lattice parameter [111,112].

For Y^3+^ and F^−^ ions that have a smaller ionic radius size compared with Ca^2+^ and OH^−^, studies revealed that after substitution, the lattice parameters and unit cell volume of *HAp* decreased [113,114]. According to another study, the use of a high concentration of 7.5 mol% Y^3+^ and 2.5 mol% F^−^ caused a decrease in relative density, but if the synthesis was performed at a temperature higher than 1300 °C, the relative density increased [115]. The samples analyzed by Raman spectroscopy confirmed that Y^3+^ and F^−^ ions used as co-doping agents for *HAp* led to perturbation in the hexagonal structure, shifting Raman bands to further bands located inside the spectra of Y/F-*HAp* [116].

The effect of use of Al^3+^ and F^−^ ions as co-substitution agents for hydroxyapatite was also studied. The samples were obtained by the precipitation method and sintered at 1100 °C. The increase in sintering time and concentration of co-doping ions led to an increased density in Al/F-*HAp* and a decrease in lattice parameters. Generally, the presence of Al^3+^ ions may have a negative effect on mechanical properties of the biomaterial and, therefore, Mg^2+^ ions are preferred instead [117].

Another study was focused on the effects of Mg^2+^ and various amounts of Ni^2+^ ions for co-doping. The samples were synthesized at 870 °C by wet chemical method and the concentration of Ni^2+^ ions was 0%, 0.6%, 1.2%, and 1.8 % at. The Ni^2+^ concentration influenced the lattice parameters and crystallinity degree, as well as the unit cell volume [118].

Using the microwave refluxing system, substitution of *HAp* with Fe^3+^ and selenite (SeO_4_^2−^) ions was also studied. Micrograph images of Fe/Se-*HAp* samples showed that the morphology was influenced by the increase in Fe^3+^ concentration [119].

**Figure 4:**
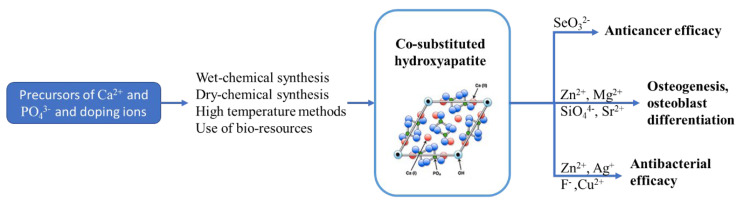
Schematic representation of synthesis methods for doped hydroxyapatite and the effect of the doping ions on anticancer, antibacterial, and osteogenesis properties [90].

Depending on the properties that are expected on the material, a combination of doping agents can be used. In Figure 4, the synthesis technology for ionic-substituted *HAp* and the effects of ions for different properties of material is presented [120]. The combined effect of the doping ions can be modified depending on the synthesis method preferred in order to obtain the desired dissolution rate of the biomaterial. The effects of co-doping on biological performance are summarized in Table 2. Sr^2+^ ions are usually used for doping hydroxyapatite to intensify osteogenesis and offset the negative effect of other ions. Bismuth is also considered to be a proper substituent for *HAp* and the co-substituted Sr/Bi-*HAp* biomaterial has high antibacterial activity against *E. coli* [121]. Cu^2+^/*HAp* samples show high antibacterial activity, but the synthesis should be performed carefully because Cu^2+^ could be toxic to human cells at high concentrations [122]. Zn^+^ has an important role in the osteogenesis process in bone remodeling. Published studies demonstrated that the toxicity level of Zn^+^ ions was increased up to 6 wt.% and the samples showed antibacterial properties against *E. coli* and *S. aureus*.

Along with Zn^+^ and Cu^2+^, studies were performed to demonstrate that Ag^+^ ions have excellent antibacterial activity. Ag^+^ ions are frequently used as a dopant agent for hydroxyapatite for antibacterial applications due to its large spectrum of antimicrobial activity. The most common use is in the medical field for vascular grafts and implants because of its antiseptic properties for wound dressing and burn creams [123]. Ag^+^ ions are widely used for antimicrobial biomaterial applications as they can influence the bacterial replication process and possible cell death [124,125].

In a similar way, Ti^4+^ was also incorporated into the structure of *HAp*, together with Zn^+^, Ag^+^, and Cu^2+^ ions using the co-precipitation method. The results were excellent for Ti/Ag-*HAp* and Ti/Cu-*HAp* which exhibit a high efficiency against *E. coli* and *S. aureus* under weak UVA irradiation due to the combination of oxidation character of O_2_^−^ radicals and antibacterial properties [126].

**Table 2:** Biological performance of various co-doping agents.

Co-Doping Agents	Synthesis Method	Biological Performance
Zn^2+^, SeO_3_^2−^ [120]	Precipitation method	Cytotoxic for BALB/c 3T3 mouse fibroblasts
Zn^2+^, Cu^2+^ [122]	Electrolytic deposition	Antibacterial properties, promoting the MC3T3-E1 osteoblasts cell viability
Zn^2+^, Sr^2+^ [127]	Hydrothermal method	Antibacterial properties, improving the cytotoxic limit of Zn^2+^, adhesion
Sr^2+^, Ag^+^ [124]	Hydrothermal method	Antibacterial properties, high osteoinductive properties, promote ALP activity in vitro
Sr^2+^, Bi^3+^ [121]	Microwave-assisted precipitation method	Antibacterial properties
Sr^2+^, F^−^ [121]	Hydrothermal method	Antibacterial properties, promote ALP activity
SiO_4_^4−^, Ag^+^ [128]	Precipitation method	Enhanced osteoconductive properties in vitro
Sr^2+^, Ag^+^, F^−^ [125]	Electrolytic deposition	Induce bone formation, high early bone integration ability

Numerous types of ions are used as substitution agents for hydroxyapatite and various studies are available to gain more knowledge on this field. On the other hand, depending on the expected properties of the synthesized biomaterial and the applications, researchers can modify the dopant, the synthesis conditions, and can choose the appropriate technique. A good progress was made through the years to obtain novel biomaterials with sustainable long-term antimicrobial properties, but the use in clinical applications is still in development stage. The use of hydroxyapatite in various combinations with other types of biomaterials in order to improve and widen biomaterial applications is further studied in this paper.

## 3. Focus on Bioactive Glasses, Bioglass^®^ 45S5

All bioactive materials have the capability to form an interfacial bond with bones or tissues, but the type of biomaterial influences the time dependence of bonding and the strength, thickness, and mechanism of bonding. Bioactive glasses have higher potential to stimulate bone regeneration compared with other types of bioactive ceramics.

### 3.1. Structure and Synthesis of Bioglass

Silica-based bioactive glasses are a group of surface-reactive glass–ceramic biomaterials. Hench et al. [20] were the first to obtain the first-generation bioglass at the University of Florida. The compound known as Bioglass^®^ (commercial name: 45S5 *BG*) is a quaternary system, composed of 45% silica (SiO_2_), 24.5% sodium oxide (Na_2_O), 24.4% calcium oxide (CaO), and 6% phosphorus pentoxide (P_2_O_5_) as weight percent, the last component being added to simulate the Ca/P ratio of hydroxyapatite [129]. The name 45S5 comes from the weight percent of silica as the network former and the 5 to 1 molar ratio of Ca to P. The paper published in 1971 by Hench presents the composition of the Bioglass and the results obtained by transmission electron microscopy (TEM), confirming the bonding to bone. Additionally, other research was conducted to test substitution of SiO_2_ with B_2_O_3_ (5–15 wt.%) and the substitution of CaO with CaF_2_ (in a concentration of 12.5 wt.%), but the substitutions did not improve the capacity of the material to form a bone bond [6]. In Table 3, the most important steps for the development of bioactive glasses and timeline are presented.

Originally, 45S5 Bioglass^®^ was produced by melt-processing of oxides with high-purity in a furnace at 1370 °C. The melt mixture is poured into water to quench, obtaining a frit. Next steps are drying and grounding, in order to obtain the appropriate particle size range. The Bioglass can be transferred into molds and rods or as-cast components can be produced [140]. This method is considered simple and suitable for higher quantities of products. However, the shortcoming of the method is the evaporation of P_2_O_5_ because of the high temperatures involved. Recent studies were focused on the sol–gel technique. This method requires a lower temperature than the traditional technique and gives the possibility to obtain a homogeneous composition [141]. Additionally, if clinical applications are considered, a method that will avoid contamination of the final product is preferred. The traditional crucible method can be replaced with a containerless technique, which allows fine control of the bioglass purity. The advantages of using the containerless technique instead of the traditional method, where the interactions of the prepared samples with the crucible wall create internal stress, are the achievement of supercooled liquids, rapid solidification, and suppression of heterogeneous nucleation [142].

### 3.2. Clinical Applications of 45S5 Bioglass^®^ and Bioceramics

Bioactive materials promote an adherent interface with tissues, sufficient to resist mechanical fracture. With excellent biocompatibility, stimulatory effects on bone cell functions and the capacity to link with bone via chemical bond and with other tissues, bioactive glass is currently used for applications in orthopedic and dental fields, to replace bones or teeth, dental devices, or as a coating for orthopedic anchors [143]. The bioactivity of Bioglass^®^ is based on its amorphous structure, but depending on the composition, the bioactivity, bioresorbability, and osteoconductivity could be modified [144]. In Table 4, the key roles of Bioglass in medical and dental fields are listed [132]. A novel discovery in this area is the use of Bioglass in preventive treatments—for prevention of dentinal pain sensitivity and for its effects on inhibition of gingivitis.

In 1985, FDA approved the first medical device based on 45S5 Bioglass^®^, commercialized under the name “Bioglass^®^ Ossicular Reconstruction Prosthesis” or “Middle Ear Prosthesis” MEP^®^, a medical device used to treat conductive hearing losses. Another medical device called Bioglass^®^-EPI was developed 30 years ago and it was used as an extracochlear percutaneous implant to the temporal bone of patients with hearing loss. Following MEP^®^ and Bioglass^®^-EPI, another bioglass-based device was commercialized under the name ERMI^®^ in 1988. The Endosseous Ridge Maintenance Implant is used in periodontal surgery [138]. In Finland, researchers developed another material starting from the composition of 45S5 Bioglass^®^, BonAlive^®^, used in the orthopedic field as a bone graft substitute. PerioGlas^®^, a particulate 45S5 Bioglass^®^ product, is now sold by NovaBone Products LLC, Florida, in more than 35 countries worldwide. With a particle size range of 90–710 µm, PerioGlas^®^ is used to regenerate bone teeth for dental intraosseous, oral, and cranio-/maxillofacial bony defects and is also used in combination with polymeric membranes. Biogran^®^, sold by BIOMET 3i, Florida, has the same composition as 45S5 Bioglass^®^, the only difference is the particle size of 300–360 µm. This material is a synthetic bone graft used for its bone regeneration effects. NovaBone^®^ (sold by NovaBone Products LLC, Alachua, FL, USA) is used since 1999 to repair bone defects in maxillofacial or orthopedic non-load-bearing sites.

Apart from the commercial forms used to repair bone and teeth defects, other types of 45S5 Bioglass^®^ were developed for oral care. NovaMin^®^, a specific kind of 45S5 Bioglass^®^, has a particle size of 18 µm and is used in toothpaste to prevent tooth hypersensitivity and for whitening treatments of teeth [145]. In Table 5, the commercial products mentioned above and their structural differences are presented.

Since 2009, endodontic biomaterials with excellent biocompatibility in vitro and low cytotoxicity, such as Biodentine, ProRoot Mineral Trioxide Aggregate (MTA), and PulpGuard, were developed and became commercially available. Several studies revealed that this new type of bioceramics composite biomaterials can enhance cell attachment and proliferation. These biomaterials are used for direct pulp capping, pulpotomy, or apexification [146,147].

**Table 5:** Schematic presentation of commercial products based on 45S5 Bioglass^®^.

Material	Composition and Structure	References
PerioGlas^®^	Contains calcium, sodium, phosphate and silica; it is a granulated form of 45S5 Bioglass^®^ Particle size: 90–710 µm	[148]
Biogran^®^	Same composition as 45S5 Bioglass^®^ (calcium, sodium, phosphate, and silica); particles of narrow size range Particle size: 300–360 µm	[149]
BonAlive^®^	Special bioglass (S53P4), consists of 53% SiO_2_, 23% Na_2_O, 20% CaO, and 4% P_2_O_5_ as weight percent Particle size: 500–800 μm	[150,151]
NovaMin^®^	Identical with 45S5 Bioglass^®^, contains only calcium, sodium, phosphate, and silica in an amorphous matrix Particle size: 18 µm	[152]

### 3.3. Derived Bioglass Bioactive Materials

Even if 45S5 Bioglass^®^ is by far one of the most investigated materials for its capability to repair damaged bone tissues, the clinical cases are vast and may differ, each one being also influenced by human metabolism, patient’s age, the severity of the injury, etc. [153]. Significant work was carried out during past years to discover which is the optimum composition of a bioglass and it has been demonstrated that the amount of SiO_2_ and Ca/P ratio can be changed in order to improve bioactivity. Researchers developed methods of incorporating various ions such as magnesium (Mg^2+^), calcium (Ca^2+^), cerium (Ce^3+^), zinc (Zn^2+^), aluminum (Al^3+^), and strontium (Sr^2+^) in order to improve bioglass characteristics. Recent studies revealed that bismuth cations (Bi^3+^) are also efficient when incorporated into bioglass lattice, with nanocomposites of bismuth-doped bioglass/graphene oxide being successfully used in bone tissue engineering [154].

It is already noted that strontium can be a substitute for calcium in bioglass structures, with the capability to improve osteoblast activity and to minimize bone fracture [155]. The bioactive coating may impart an antibacterial effect to the environment, not only between bone and implant. Studies have revealed that a small concentration of Sr^2+^ (2.5 mol%) manifests antibacterial properties against *S. aureus, E. coli,* and *P. gingivalis*.Ca^2+^ and Sr^2+^ have similar ionic structures, hence the incorporation of strontium ions would modify the dissolution of the bioglasses. The addition of Ca^2+^ in a high amount can lead to a decrease in osteoblast viability. Studies in vitro and in vivo revealed that Sr^2+^ positively influenced osteoblast proliferation and bone formation [156].

Zinc was also added into the bioglass structure due to its beneficial properties on bone formation in vitro and in vivo [157,158,159]. Raman spectra, EDX microscopy, and studies performed on cell culture revealed that the incorporation of Zn^2+^ into the bioglass system is beneficial for cell attachment. SiO_2_-CaO-P_2_O_5_-ZnO biomaterial is highly bioactive and biocompatible in vitro, and the substitution of Zn^2+^ enhances early cell proliferation and promotes differentiation [129].

Another possibility for substitution is the incorporation of Al^3+^/Sr^2+^ ions in the same amount (2 mol%). Tests revealed that the bioactivity was maintained, even if the alumina influenced the degradation of bioglass. No changes in the bioglass lattice were reported. Moreover, the studied samples exhibited an antibacterial effect against *E. coli* strains. The combined effect of antibacterial activity and biocompatibility promotes bioactive glasses to be used for bone regeneration [157].

The addition of iron oxide (Fe_2_O_3_) into the bioglass structure reveals the possibility to use the material for magnetic induction hyperthermia treatment of cancer. Specific types of bioglasses (SiO_2_-CaO-Fe_2_O_3_, SiO_2_-CaO-Fe_2_O_3_-B_2_O_3_-P_2_O_5_, and SiO_2_-Al_2_O_3_-Fe_2_O_3_-P_2_O_5_-Li_2_O) were synthesized by controlled crystallization. Depending on the amount of iron oxide from the samples, studies were made to analyze the evolution of magnetic properties and the crystallite size [160].

Recent development was made for incorporating luminescent materials into the bioglass structure in order to obtain nanoparticles that can be used for biological labelling applications and drug delivery systems [161,162]. The possibility to have luminescence characteristics would be helpful for understanding bioglass degradation in vitro. Er^3+^ and Yb^3+^ were used as co-doping agents for bioglass using a containerless method in an aerodynamic levitation furnace. The tests performed on the Er^3+^/Yb^3+^ co-doped bioglass samples exhibit visible up-conversion luminescence and the cell proliferation assay suggests the biocompatibility and bioactivity of the material for cell proliferation, similar with the pure bioglass [163].

The substitution with other rare earth elements, Tb^3+^/Yb^3+^, into the bioglass structure was studied to obtain a multicomponent bioglass with luminescent properties. The elemental composition of 45S5 Bioglass^®^ was standard and the amount of SiO_2_ was increased to 50 wt.%, with the Ca/P ratio being maintained at 4:1 as in 45S5 Bioglass^®^ and then modified at 1.67. The most important factors for network connectivity and structural properties of bioglass are concentration, the method of synthesis, and temperature. The amorphous nature of bioglass until 700 °C was confirmed by XRD analysis and the in vitro bioactivity test confirmed that all samples of Tb^3+^/Yb^3+^ co-doped bioglass exhibit good apatite-forming ability [164].

## 4. Hydroxyapatite/Bioglass 45S5 Composites

In order to develop a new hydroxyapatite/bioglass composite, researchers should have the proper information regarding the structure, properties, and applications of the starting biomaterials. This information is summarized in Table 6 [165,166]. Bioactive glass manifests advanced properties in comparison with hydroxyapatite. On a study conducted on rabbits, researchers reported that granules of 45S5 Bioglass^®^ can promote bone proliferation more easily than *HAp*, with 45S5 Bioglass^®^ being considered osteoinductive due to its capability to support bone growth together or away from the bone–implant interface [167].

Composites containing bioactive glass/hydroxyapatite are extensively studied due to their applications for regeneration and repair of bone tissue. Some studies were focused on the possibility to use bioglass/hydroxyapatite (*BG/HAp*) composite as a coating on titanium substrate. Samples were prepared via the electrophoretic deposition method (EPD), a simple, versatile technique with good cost-effectiveness. The EPD method is based on the movement of charged particles toward the working electrode by applying an electric field [168]. According to Khanmohammadi et al. [169], the combination of 50% *BG*/50% *HAp* exhibits high bioactivity and maximum bonding strength, which will shorten the bioactive fixation of the implant in medical use. The tests performed on the samples shows that the pure bioglass exhibits the lowest bonding strength in comparison with the samples where *HAp* was added.

Another study reported the synthesis of bioglass by the sol–gel method, followed by the synthesis of bioglass/hydroxyapatite biomaterial through a traditional technique, heated in a furnace up to 1000 °C. Different concentrations of hydroxyapatite were used. The bioactivity of *BG/HAp* material was determined by in vitro tests. The synthesized biomaterial exhibits high bioactivity property demonstrated by FTIR and SEM analysis [170].

Rizwan et al. [171] reported the use of Spark plasma sintering method (SPS) to obtain HAp/BG composite scaffold materials. SPS technique is a non-conventional method used to obtain composites at low values of pressure and temperature in a short period of time at 1000 °C temperature. The benefits of the method are the easiness of controlling parameters, possibility to obtain amorphous or nanocrystalline phases and to avoid thermal decomposition. The content of hydroxyapatite was in the range of 0–30 wt.%. After the evaluation of prepared samples, it was demonstrated that the SPS sintering method can be used for the *HAp*/*BG* composite, showing no drastic reactions between components.

**Table 6:** Presentation of general information about hydroxyapatite and 45S5 Bioglass^®^ [165,166].

Information about Material		Material	
		Hydroxyapatite	45S5 Bioglass^®^
Composition		Ca_10_(PO_4_)_6_(OH)_2_	45 wt.% SiO_2_, 6 wt.% P_2_O_5_, 24.5 wt.% CaO, and 24.5 wt.% Na_2_O
Structure		Crystalline, hexagonal symmetry	Amorphous
Average particle size		10 nm and 500 nm	Commercial products have different particle size, from 18 µm to 800 µm
Molar mass (g/mol)		502.31	-
Density (g/cm^3^)		3.16	2.7
Melting point (°C)		1614	Approximately 1100–1200 [172]
Solubility in water		Poorly soluble in water	More soluble than other bioactive materials such as *HAp*
Mechanical properties	Compression strength (MPa)	>400	Approximately 500
	Tensile strength (MPa)	Approximately 40	42
	Elastic modulus (GPa)	Approximately 100	35
	Fracture toughness (MPa$\sqrt{m}$)	Approximately 1.0	0.5 to 1
Uses and advantages		Desirable bone replacement material due to high hardness and high bioactivity, which accelerate early stage of bone growth	Repair of bone injuries or defects due to high biocompatibility, bioactivity, and osteoconductivity Applications in tissue engineering and tooth enamel reconstruction with high antibacterial resistance
Antibacterial effect		Low antibacterial properties, doping bacteriostatic ions such as Sr, Zn, Ce, and Ag are used for substitution	Effective against a wide selection of aerobic and anaerobic bacteria [173]

After analyzing the existing literature and current research we can conclude that the hydroxyapatite/bioglass composite biomaterials have the advantages of both components. Pure bioglass and pure hydroxyapatite show minimum bonding strength, while the *HAp*/*BG* composite shows increased bonding strength at 50 wt.% of components. Even if *HAp* has excellent biocompatibility, the bioactivity is improved for composites with bioactive glass, when a silicate hydroxyapatite may be produced. Hydroxyapatite shows low antibacterial property which leads to investigations focused on modifying doping bacteriostatic ions. Studies demonstrated that 45S5 Bioglass^®^ is efficient against *S. aureus*, *S. epidermidis*, and *E. coli*. The combination of hydroxyapatite and bioglass particles confers special characteristics such as antibacterial effect, bioactivity, and mechanical properties.

## 5. Conclusions and Future Perspectives

Since calcium phosphates were proposed for the first time to be used in the biomedicine field, multiple formulations have been developed and some have been successfully used in clinical applications due to *CaPs* capacity to substitute, repair, or regenerate bones and teeth and also for drug delivery systems. *CaPs* have become one of the most used classes of biocompatible and biodegradable materials. Hydroxyapatite offers excellent biocompatibility, bioactivity, low hardness, low compressive strength, and low density, while Hench’s 45S5 Bioglass^®^ has very low degradation and excellent bioactive properties. Even if numerous studies have been focused on developing a novel biomaterial with improved properties, only a limited number of studies provided in vitro and in vivo biocompatibility of substituted and co-substituted hydroxyapatite. A newer approach is the incorporation of multiple ions or another type of biomaterial and/or bioceramics into the structure of hydroxyapatite to form new types of composites with broader applications. With this aim, bioactive glasses are used in combination with hydroxyapatite for prevention of tissue damage, technology that is in continuous expansion. The bioactivity and the mechanical strength of bioglass are improved when it is reinforced with hydroxyapatite and, even more, with a combination of hydroxyapatite/other doping ions. Studies have been conducted to identify a different approach for the synthesis method with the appropriate composition of phases.

Because of the progress that has been made in material science and chemical industries, new perspectives for the use of CaPs appeared and one of the recent trends is using calcium phosphates in the cosmetic field as an alternative to potentially dangerous ingredients. Some general trends can also be anticipated. First of all, we expect that the scientific work in this field will be focused on improving *CaPs* properties and efficacy using various ion-doping methods, and not only based on hydroxyapatite, but also using different *CaPs* phases with different properties. Secondly, if we are considering the nanomedicine field, we should highlight the use of *CaP* nanoparticles for drug delivery. Ultimately, we anticipate that the principal sources of *CaPs* will be biogenic sources and byproducts, as sustainability is a keyword nowadays.

## Figures and Tables

**Table 1:** Comparison between hydroxyapatite nanostructures of different sizes and morphology depending on the synthesis route.

Synthesis Technology	Advantages	Morphology and Size
Co–precipitation [40,41,42,43]	The most efficient method at room temperatureThe particle size depends on the pH and ionic strength of the reaction media	Rod, sphere, fiber, tube, filament, whisker, flower Size: 3 nm–1000 µm
Sol–gel method [40,44]	The mixing of reactant at molecular level may improve the chemical homogeneity	Sphere, needle, tube, rod, filament, platelet Size: 3 nm–1000 µm
Hydrothermal method [40,45,46,47]	Enhanced solubility of precursors	Irregular, rod, needle, filer, whisker, feathery structures Size: 5 nm–8 µm
Sonochemical [40,48,49,50]	The possibility of producing monodispersed nanoparticles of different shapes	Sphere, filament, tube, rod Size: 5 nm–1000 µm
Thermal decomposition [40,51,52,53]	Method that allows a good particle size control and good crystallinity	Flake, plate, sheet, formless particles Size: 5 nm–200 µm
Combustion [40,54,55]	A quick method with high purity and a single step procedure	Sphere, oval, irregular spherical shapes Size: 5 nm–200 µm
Solid-state dry synthesis [40,56,57]	Particles with a well-crystallized structure	Irregular, rod, needle, whisker Size: 5 nm–1000 µm
Biosources [58,59,60,61]	Ecofriendly technique to transform waste from various natural sources to wealth	Different shapes: flakes, plate, rod, tubular shape, etc. Size: 10 nm–2000 µm

**Table 3:** Development of 45S5 Bioglass^®^.

Year	Development Steps
1969	45S5 Bioglass^®^ was discovered at the University of Florida
1971	Based on the research activity, bonding of bone to bioactive glasses and glass–ceramics study was published [20,130]
1975	Bioglass-coated alumina was used for bone bonding on sheep hip implants [131]
1977	Bioglass coating on metals and alumina ceramics was patented [132]
1981	Discovery of soft connective tissue bonding to 45S5 Bioglass^®^ [133]
1988	Bioglass is approved by FDA to be used on ERMI implant [132,134,135]
1991	Sol–gel synthesis was investigated for bioactive gel-glasses [136,137]
1993	PerioGlas^®^ was approved by FDA to be used for bone and dental repair [138]
1996	PerioGlas^®^ (bioglass used for bone grafting) is used in tooth extraction and alveolar augmentation [132]
1999	TheraSphere^®^ was approved by FDA to be used for cancer treatment [138]
2000	Studies were made to examine if 45S5 Bioglass^®^ can be used to control osteoblast cell cycles [139]
2004–2005	NovaMin^®^ (particular type of 45S5 Bioglass^®^) is developed for use as repair agent in toothpaste [132]
2010	Cardiac tissue engineering [138]
2012	Spinal cord repair [138]
2018	TheraSphere^®^ is used for liver metastatic colorectal carcinoma [138]

**Table 4:** Orthopedics, cranial–facial and dental–maxillofacial products based on 45S5 Bioglass^®^ [132].

Medical Field	Applications
Orthopedics products	Trauma: Fractures of long bones (alone or with internal fixation) Repair of femoral Tibial plateau fracture Arthroplasty: Filler around implants Impaction bone grafting Spine fusion: Posterolateral lumbar spinal fusion Recovery of bones after tumor removal
Cranial–facial products	Cranioplasty: Facial reconstruction Augmentation of alveolar ridge General dental/oral improvement: Sinus lift surgery Cutting or reshaping of bones Repair of periodontal defects
Dental–maxillofacial products	Toothpaste and treatments to prevent hypersensitivity Obliteration of frontal sinus Direct and indirect pulp capping Repair of orbital floor fracture
